# Life Course Associations of Sibling Relationships and Cognitive Functioning in Late Adulthood

**DOI:** 10.1093/geroni/igaf122.2108

**Published:** 2025-12-31

**Authors:** Sara Moorman, Jooyoung Kong, Gina Lee, Michal Engelman

**Affiliations:** Boston College, Chestnut Hill, Massachusetts, United States; University of Wisconsin–Madison, Madison, Wisconsin, United States; University of Wisconsin–Madison, Madison, Wisconsin, United States; University of Wisconsin-Madison, Madison, Wisconsin, United States

## Abstract

**Background/objective:**

Relying on the linked lives concept within the life course perspective, the current study examined the lifelong associations of sibling relationships and their effects on cognitive functioning in late adulthood.

**Methods:**

Using data from the sibling cohort of the Wisconsin Longitudinal Study, we estimated a mediational model, examining the effects of childhood sibling interactions and adverse childhood experiences (ACEs), as proxy measures for the parent-child relationship, on cognitive functioning in late adulthood through adult sibling closeness and contact. Respondents’ cognitive functioning was assessed using the Telephone Interview for Cognitive Status-modified (TICS-m) when they were in their early 80s, on average. We conducted multilevel structural equation modeling.

**Results:**

Positive childhood sibling interactions (e.g., hugging, helping) were associated with increased adult sibling closeness and contact, whereas higher cumulative ACEs were associated with decreased adult sibling closeness and contact. We found significant indirect associations: (a) positive sibling interactions during childhood were associated with more frequent sibling contact across respondents’ mid-50s through early 70s, which was in turn associated with higher TICS-m scores in their early 80s; (b) higher cumulative ACEs were associated with less frequent sibling contact, which was in turn associated with lower TICS-m scores.

**Conclusions:**

The results suggest that childhood experiences within the family of origin may have lasting effects on adult sibling relationships and the cognitive functioning of older adults. This study highlights the potential cognitive benefits of frequent contact with adult siblings, underscoring the importance of nurturing sibling relationships throughout one’s life.

